# Microbiota DNA isolation, 16S rRNA amplicon sequencing, and bioinformatic analysis for bacterial microbiome profiling of rodent fecal samples

**DOI:** 10.1016/j.xpro.2022.101772

**Published:** 2022-10-21

**Authors:** Chloe J. Love, Carolina Gubert, Saritha Kodikara, Geraldine Kong, Kim-Anh Lê Cao, Anthony J. Hannan

**Affiliations:** 1 The Florey Institute of Neuroscience and Mental Health, University of Melbourne, Parkville, VIC 3010, Australia; 2 Department of Anatomy and Physiology, University of Melbourne, Parkville, VIC 3010, Australia; 3 Melbourne Integrative Genomics, School of Mathematics and Statistics, University of Melbourne, Parkville VIC, 3010, Australia

**Keywords:** Bioinformatics, Single Cell, Cancer, Health Sciences, Genetics, Genomics, RNAseq, High Throughput Screening, Microbiology, Model Organisms, Molecular Biology, Systems biology

## Abstract

Fecal samples are frequently used to characterize bacterial populations of the gastrointestinal tract. A protocol is provided to profile gut bacterial populations using rodent fecal samples. We describe the optimal procedures for collecting rodent fecal samples, isolating genomic DNA, 16S rRNA gene V4 region sequencing, and bioinformatic analyses. This protocol includes detailed instructions and example outputs to ensure accurate, reproducible results and data visualization. Comprehensive troubleshooting and limitation sections address technical and statistical issues that may arise when profiling microbiota.

For complete details on the use and execution of this protocol, please refer to Gubert et al. (2022).

## Highlights

• Profile bacterial populations of the gastrointestinal tract using rodent fecal samples
• Initial steps include collection of fecal samples and isolation of genomic DNA
• Further steps include 16S rRNA gene V4 region sequencing and bioinformatic analyses

## Abstract (editor highlights)

Publisher’s note: Undertaking any experimental protocol requires adherence to local institutional guidelines for laboratory safety and ethics.

## Graphical abstract

**Figure:**
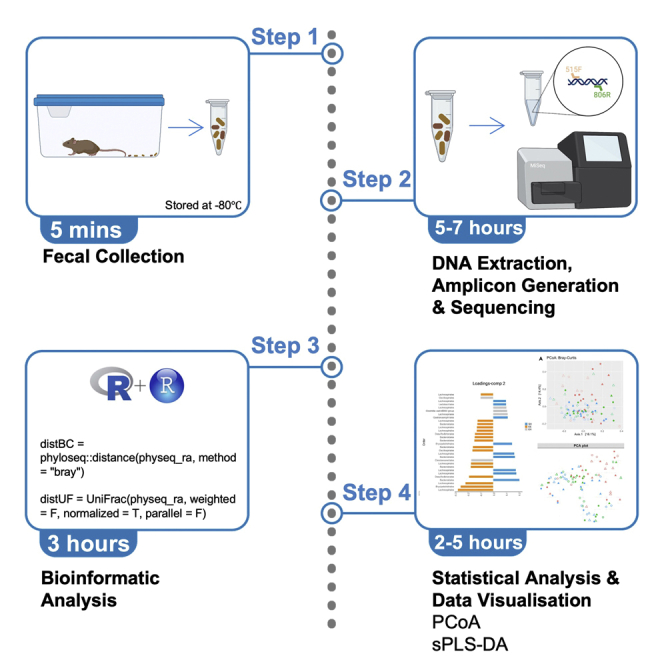

## Before you begin

1. Clearly label all tubes for fecal collection.
2. Ensure you have access to the appropriate versions of R and the relevant packages.
3. Ensure all mice are housed in the same experimental room with the appropriate light/dark cycles and controlled for temperature and humidity.

### Institutional permissions

Experiments were all approved by the Florey Institute of Neuroscience and Mental Health Animal Ethics Committee, AEC project number 19-012-FINMH and conducted according to the National Health and Medical Research Council animal research guidelines. Every effort was made to minimize the number of animals used and ensure ethical treatment. All experiments conducted using animals require permissions and approval from the relevant institutions.

## Key resources table

**Table:** 

REAGENT or RESOURCE	SOURCE	IDENTIFIER
**Biological samples**		

Mouse feces (4–8 pellets)	Mouse	

**Critical commercial assays**		

QIAamp PowerFecal Pro DNA kit	QIAGEN	Catalogue number 51804
ZymoBIOMICS Microbial Community Standard	Zymo Research	Catalogue number D6300
Qubit dsDNA HS Assay kit	Thermo Fisher Scientific	Catalogue number Q32851
Platinum Hot Start PCR master mix (2×)	Thermo Fisher Scientific	Catalogue number 13000013
Ampure XP Reagent	Beckman Coulter	Catalogue number A63881
MiSeq Reagent Kit v2, 300 cycles	Illumina	Catalogue number MS-102-2002
Nuclease-free water	Sigma-Aldrich	7732-18-5
515 forward primer with the following sequence whereby XXXXXXXXXXXX contains the unique Golay barcodes: AATGATACGGCGACCACCG AGATCTACACGCT XXXXXXXXXXXX TATGG TAATTGTGTGYCAGCMGCCGCGGTAA	IDT	Custom
806 reverse primer with the following sequence: CAAGCAGAAGACGGCATACGAGATAGTCAGCCAG CC GGACTACNVGGGTWTCTAAT	IDT	Custom
Read 1 sequencing primer: TATGGTAATT GT GTGYCAGCMGCCGCGGTAA	IDT	Custom
Read 2 sequencing primer: AGTCAGCCAG CC GGACTACNVGGGTWTCTAAT	IDT	Custom
Index sequencing primer: AATGATACGGCG ACCACCGAGATCTACACGCT	IDT	Custom

**Deposited data**		

The datasets and metadata related to this protocol have been deposited in the NCBI Sequence Read Archive	This protocol	BioProject Number PRJNA770470
The reproducible R code and report for the statistical analysis	This protocol	https://github.com/SarithaKodikara/Gene_environment_gut_interactions_in_Huntington-s_disease

**Experimental models: Organisms/strains**		

Male & female mice, age 12 weeks, strain R6/1 B6.Cg-Tg(HDexon1)61Gpb/J	The Jackson Laboratory	JAX: 0006471

**Software and algorithms**		

R (Version 4.1.0)	R Development Core Team	https://www.r-project.org/
Prism 9 (Version 9.3.0)	GraphPad	https://www.graphpad.com/scientific-software/prism/
PhiX Control v3	Illumina	https://sapac.illumina.com/products/by-type/sequencing-kits/cluster-gen-sequencing-reagents/phix-control-v3.html

**Other**		

Personal Protective Equipment	Laboratory Supplied	Gloves, face mask, gown
Paper Towel	Laboratory Supplied	
80% & 100% Ethanol	Thermo Fisher Scientific	T08204K7, A4094
Dry Ice	Laboratory Supplied	
Esky or Polystyrene Cooler	Medisa	611115
Standard mouse cage base and wire lid (34 cm × 16 cm × 16 cm)	Wiretainers	MB1
5 mL Eppendorf Tubes	Sigma-Aldrich	Z688223
0.5 mL thin-wall, clear PCR tubes	Axygen	PCR-05-C
Needle 30G × 1/2″ 13 mm	Admiral Medical Supplies	TE3013
Biosafety Cabinet	Laboratory Supplied	
Precellys 24 tissue homogenizer	Bertin Instruments	P000669-PR240-A
NanoDrop™	Thermo Fisher Scientific	ND-ONEC-W
Pipettes, 1–500 μL	METTLER TOLEDO	L-10XLS+, L-100XLS+, L-200XLS+, L-2000XLS+
BRAND pipette tips (2–200 μL)	Merck	Z740030
Thermal cycler	Thermo Fisher Scientific	Automated Thermal Cycler (ATC)
0.2 N NaOH (less than a week old)	Thermo Fisher Scientific	60-026-26
MiSeq sequencing platform	Illumina	https://sapac.illumina.com/systems/sequencing-platforms/miseq.html

## Step-by-step method details

### Fecal collection

**Timing: 5 min per mouse**

Mouse fecal collection is to be completed in a sterile environment and as quickly as possible, to avoid potential microbial contamination. Ensure appropriate PPE is worn and all items used in the collection process are sterilized to minimize sample contamination.

1. Assemble cage base with lid and label each cage with appropriate mouse number prior to beginning fecal collection.
2. Spray each cage with 80% ethanol and wipe with paper towel. Wait until the cage is completely dry as an additional sterilization measure.
3. Place each mouse into the appropriately labeled clean cage for up to 5 min or until the mouse has excreted 4–8 fresh fecal pellets or a minimum of 35 mg of feces.
4. Return the mouse to the home cage.
5. Using a fresh needle for each collection, use the needle tip to carefully collect the pellets and place into an appropriately labeled 5 mL Eppendorf tube.

***Note:*** Do not collect fecal pellets that have been in contact with urine, see troubleshooting 1.

6. Place the Eppendorf tube immediately into a cooler box with dry ice, before storing at −80°C until further processing.

***Note:*** All cages are sterilized using a commercial cage and rack washer and commercially available chemicals. The wash cycle runs for 240 s at 55°C using TP Alka detergent followed by a dripping cycle for 30 s and neutralization for 4 s using TP Acid. Neutralization is followed by a rinse cycle for 20 s at 82°C, steam sanitization and an exhaust cycle for 60 s at 45°C.

***Note:*** Immediately freezing and storing fecal samples at −80°C is considered best practice for preserving microbial composition (Fouhy et al., 2015; Wu et al., 2010). Bacterial species remain viable for up to 10 years when stored at −80°C (Simione et al., 1991).

### DNA extraction from fecal pellets

**Timing: 5–7 h for 94 samples and 2 controls**

This step aims to extract genomic DNA from the previously collected fecal samples. Ensure appropriate PPE is worn during the extraction process.

7. Extraction of genomic DNA in fecal samples:
  a. In a biosafety cabinet, take ∼25 mg of mouse fecal sample and prepare the genomic DNA using the QIAamp PowerFecal Pro DNA kit as per manufacturer’s instructions (QIAGEN).
  b. For the positive control, thaw and vortex the ZymoBIOMICS Microbial Community Standard (Zymo Research) to ensure even mixing. Take 30 μL of the microbial standard and add to a PowerBead tube containing C1 buffer provided in the kit.
  c. For the negative control, add in 50 μL of nuclease-free water to a PowerBead tube containing C1 buffer provided in the kit.
  d. Perform bead beating at 5,000 rpm for 30 s on Precellys 24 tissue homogenizer (Bertin Instruments) or equivalent homogenizer to ensure complete lysis of the sample.
  e. Follow the manufacturer’s instructions for the remaining steps and elute the extracted DNA with 85 μL of nuclease free water. Troubleshooting 2.
8. Quantify the extracted DNA using Qubit dsDNA HS assay kit, Quant-iT PicoGreen dsDNA Assay kit or equivalent.
9. Assess the purity of the extracted DNA by measuring the A260/230 and A260/280 ratio using Nanodrop.

***Note:*** This protocol describes the steps for the processing of fecal samples using QIAamp PowerFecal Pro DNA kit. Other kits that could be used for manual preparation include QIAGEN DNeasy PowerSoil Kit, or QiaSymphony PowerFecal Pro DNA kit. It is important to choose kits specifically for extracting genomic DNA from fecal or soil material to enable efficient lysis and to eliminate inhibitors of downstream sequencing applications that are commonly found in fecal samples.

### 16S amplicon library generation

**Timing: 5–7 h when running triplicate PCR reactions in parallel**

This step aims to generate the 16S sequencing library targeting the V4 hypervariable region of the 16S rRNA for sequencing following the protocol outlined by Caporaso et al. (2018) for the Earth Microbiome Project. The Golay barcodes for demultiplexing are located on the forward primer 515F. Ensure appropriate PPE is worn during this process.

***Note:*** PCR products can contaminate reagents, instruments and genomic DNA samples; please see troubleshooting 3, for potential solutions.

10. Prepare the 515F and 806R primer:
  a. Resuspend the lyophilized primer in nuclease-free water for a stock concentration of 100 μM.
  b. Incubate at room temperature for 2 min.
  c. Vortex for 10 s and then centrifuge the primers at a speed of 15,000 g for 30 s.
  d. Dilute the primers to a working concentration of 10 μM.
11. Perform PCR amplification of 16S V4 rRNA:
  a. Prepare the PCR reaction master mix containing the following volumes per reaction and multiply by three to perform PCR reactions in triplicates:

**Table:** 

Reagent	Amount for 1 reaction	Amount for 1 sample (3 reactions)
Platinum Hot Start PCR master mix (2×)	10 μL	30 μL
806 Reverse primer (10 μM)	0.5 μL	1.5 μL
Nuclease-free water	13 μL	39 μL
Total reaction volume	23.5 μL	70.5 μL
  b. Aliquot the master mix according to the number of reactions into thermocycle-compatible tubes or 96-well PCR plate.
  c. Add in 0.5 μL of the Golay-barcoded 515 forward primer into each tube or well. Each triplicate reaction of the same sample should have the same Golay-barcoded 515 forward primer and each sample should have a unique Golay-barcoded 515 forward primer.
  d. Add in 1 μL of DNA template into each reaction.
  e. The final reaction should contain the following:

**Table:** 

Reagent	Amount for 1 reaction
DNA template	1 μL
Platinum Hot Start PCR master mix (2×)	10 μL
515 Forward primer (10 μM)	0.5 μL
806 Reverse primer (10 μM)	0.5 μL
Nuclease-free water	13 μL
Total reaction volume	25 μL
  f. Setup the thermocycler conditions as follows and run the PCR:

**Table:** 

Steps	Temperature	Time	Cycles
Initial Denaturation	94°C	3 min	1
Denaturation	94°C	45 s	35 cycles
Annealing	50°C	60 s	
Extension	72°C	90 s	
Final extension	72°C	10 min	1
Hold	10°C	Forever

    **Pause point:** The PCR cycle will take approximately 2 h 45 min and the PCR reactions can remain on the thermocycler until the next step.
12. Pooling and normalizing amplicon libraries:
  a. Pool the triplicate PCR reactions for each sample into a single volume of 75 μL. Run the amplicon libraries on an agarose gel to verify the presence of PCR product with the expected size of ∼390 bp.
  b. Quantify the amplicons using Qubit HS assay kit, Quant-iT PicoGreen dsDNA Assay kit or equivalent. The expected ranges for positive and negative control are 10–30 ng/μL and 0–2 ng/μL respectively. Fecal samples are expected to generate an amplicon concentration of 10–30 ng/μL. Troubleshooting 4.
  c. Combine 240 ng of each sample into a single tube. Pool 2 μL of the negative control and 10 μL of the positive control in the final tube.
13. Clean-up the amplicon library pool using Ampure XP beads (Beckman Coulter).
  a. Allow the beads to come to 23°C and vortex to resuspend before usage.
  b. In a clean Eppendorf tube, add 200 μL of the resuspended Ampure XP beads to 400 μL of amplicon pool and repeat until all the pool has been transferred.
  c. Mix by inverting the tube 10 times.
  d. Incubate at 5 min at 23°C.
  e. Pellet the beads on a magnetic rack for approximately 2 min or until the solution turns clear and a visible bead is formed close to the magnet.
  f. Prepare 80% v/v ethanol in nuclease-free water.
  g. Wash the beads:
    i. Pipette off the supernatant and discard.
    ii. Wash the beads with 500 μL of freshly prepared 80% v/v ethanol without disturbing the pellet.
    iii. Pipette off the 80% v/v ethanol and discard.
  h. Repeat the wash one more time.
  i. Pulse centrifuge the tube and replace the tubes on the magnetic rack.
  j. Pipette off residual ethanol and allow the pellet to dry for ∼30 s. Do not overdry the pellet to the point of cracking, a good rule of thumb is that the pellet should be wet but not shiny.
  k. Remove the tube from the magnetic rack and resuspend pellet in 200 μL nuclease-free water. Incubate at room temperature for 2 min.
  l. Return the tubes to the magnetic rack until the solution is clear. Remove and retain 200 μL of eluate from all tubes into a single tube.
    i. Quantify the pool using Qubit HS assay kit, Quant-iT PicoGreen dsDNA Assay kit or equivalent. The pool should be between 0.5–5 ng/μL. Given that the expected amplicon size is 390 bp, and dsDNA is 660 g/mol/bp, determine concentration of the pool in molarity as below:

Conc. in nM = $\frac{X\;ng/\mu l}{\left(390\right)\left(660\right)}\;$ x 10^6^

14. If a pool is less than 2 nM, it may be required to repeat pooling and Ampure XP concentration. Otherwise, dilute the amplicon pool to 2 nM.

### 16S amplicon library sequencing

**Timing: 0.5–1 h of preparation, ∼24 h on the MiSeq instrument**

This step aims to sequence the 16S amplicon library on the MiSeq platform using MiSeq v2 Reagent kit 2 × 150 bp (300 cycles) (Illumina, San Diego, California, USA). Ensure appropriate PPE is worn during the sequencing process.

15. Prepare the 16S Read 1, Read 2 and Index sequencing primers:
  a. Resuspend the lyophilized primer in nuclease-free water to 100 μM.
  b. Incubate at room temperature for 2 min.
  c. Vortex for 10 s and then centrifuge the primers at a speed of 15,000 g for 30 s.
16. Thaw the MiSeq v2 reagent cartridge in a water bath at room temperature for at least 1 h before usage.
17. Denature and dilute the libraries to 6.5 pM:
  a. Add 10 μL of freshly diluted 0.2 N NaOH to 10 μL of 2 nM library in a clean microfuge tube.
  b. Mix by pipetting gently 10 times.
  c. Incubate for 5 min at room temperature.
  d. Quench the reaction with 980 μL of pre-chilled HT1 provided in the kit, vortex and put on ice.
  e. Further dilute the sample to 6.5 pM by adding 325 μL of the reaction from the previous step to 675 μL of HT1 in a new microfuge tube.
  f. Mix and chill until use.
18. Denature and dilute PhiX Control v3 (Illumina) to 6.5 pM:
  a. Add 2 μL of 10 nM PhiX and 8 μL nuclease-free water to a new microfuge tube.
  b. Add 10 μL 0.2 N NaOH to the tube.
  c. Mix by pipetting gently 10 times.
  d. Incubate for 5 min at room temperature.
  e. Quench with 980 μL of pre-chilled HT1 provided in the kit, vortex and put on ice.
  f. Further dilute the PhiX by adding 81.2 μL of the reaction from the previous step to 168.8 μL HT1 in a new microfuge tube.
  g. Mix and chill until use.
19. Mix the reagents in the MiSeq cartridge by inverting 5–10 times and to ensure all reagents have defrosted. Gently tap down the cartridge to settle the reagents to the bottom of the well.
20. Load the sequencing primers and library onto the MiSeq cartridge. Pierce wells with a clean 1 mL pipette tip and add:
  a. To well 12: 4 μL of Read 1 primer (100 μM).
  b. To well 13: 4 μL of Index primer (100 μM).
  c. To well 14: 4 μL of Read 2 primer (100 μM).
  d. To well 17: Prepare the final loading sample with 15% PhiX by adding 850 μL of 6.5 pM sample and 150 μL of 6.5 pM PhiX Control in a clean microfuge and load 600 μL into the well.
21. Run the sequencing on the MiSeq instrument using either Base Space or sample sheet. Troubleshooting 5.

***Note:*** To ensure that there are at least 100,000 sequencing reads per sample, which is adequate for microbiome profiling, no more than 192 samples should be multiplexed per sequencing run given the MiSeq output of 20 million reads.

***Note:*** 10%–15% of the high diversity PhiX spike-in is commonly used for best results when sequencing 16S libraries given its low diversity.

### Bioinformatic analysis & data pre-processing

**Timing: 3–5 h including data visualization**

This step aims to generate amplicon sequence variants (ASVs), taxonomic classification of ASVs, calculation of alpha and beta diversity and multivariate statistical analysis. R programming was used. Specific to this study we analyzed results based on sex and housing groups. Computational resources used included a 3.1 GHz Dual-Core Intel Core i5 processor and 16 GB 2,133 MHz LPDDR3 memory.

***Note:*** All statistical and pre-processing analyses were performed using R software (version 4.1.0), with the use of R packages biomformat V1.20.0, dada2 V1.20.0, Phyloseq V1.36.0, ggpubr V0.4.0, ggplot2 V3.3.6, mixOmics V6.16.3, nlme V3.1-158 and vegan V2.6-2. The Qiita platform was used for processing the raw FASTQ data, however the QIIME2 platform would be a suitable alternative.

As in Gubert et al. (2022), we present the specific steps to investigate sex and housing groups using R and graphical outputs. The R code below can be modified appropriately for similar statistical analyses, and for further improved analyses.

22. Process Illumina MiSeq sequence raw FASTQ data using Qiita for quality control, demultiplexing sequences and trimming to generate ASVs.

***Note:*** Qiita has built in quality control. Default options can be used to run the pre-processing pipeline. Please refer to the Qiita Processing Data page for relevant quality scores.

23. Load all required libraries into R.

> library(biomformat)

> library(dada2)

> library(phyloseq)

> library(ggpubr)

> library(ggplot2)

> library(mixOmics)

> library(nlme)

> library(vegan)

> library(DECIPHER)

> library(phangorn)

> library(gghighlight)

> library(dplyr)

24. Read and pre-process the data.
  a. Read the ASV count table saved as an ‘.biom’ file in the data folder using ‘biomformat’ R package and filter out the brain samples that were included in the biom file.
    ***Note:*** The brain samples included in the .biom file were used for a different study.
    > raw_data<-read_biom("data/DMGPR00078.biom")
    ###Converte the data into matrix format###
    > count_data<-as(biom_data(raw_data),"matrix")
    ###Order the column names to filter brain samples###
    > count_data<-count_data[,order(colnames(count_data))]
    ###Filter out the counts related to brain samples (i.e., last 23 samples were removed###
    > gut_count_data<-count_data[,-(c(96:118))]
    ###Filter ASVs that have 0 across all samples (i.e., ASVs related to brain samples)###
    > filtered_gut_counts<-gut_count_data[rowSums(gut_count_data[,])>0,]
    ### Read sample information and convert qualitative variables into factors###
    > sample<-read.csv("Data/Gut sample information (DAMG_FM009).csv")[,-c(1:3)]
    > sample$Genotype<-factor(sample$Genotype, levels=c("WT","HD"))
    > sample$Housing <-factor(sample$Housing, levels = c("SH","EE","EX"))
    > sample$Sex<-factor(sample$Sex, levels=c("Female", "Male"))
    > sample$Box <-factor(sample$Box, levels = c("1","1.1","2","2.1","3","3.1","5","5.1","6","6.1","7","7.1","9","9.1","10","10.1","11","11.1","13","13.1","14","14.1","15","15.1","17","17.1","18", "18.1","19","19.1","21","21.1","22",
    "22.1","23","23.1"),labels =c("A","a","B","b","C","c","D","d","E","e","F","f","G","g","H","h","I","i","J","j","K","k","L","l","M","m","N","n","O","o","P","p","Q","q","R","r"), ordered = TRUE)
    ### Create a new qualitative variable by concatenating two variables###
    > sample$Sex_Geno<-factor(paste0(sample$Sex,"_",sample$Genotype))
    > sample$Housing_Geno<-factor(paste0(sample$Housing,"_",sample$Genotype))
  b. Use the reference database ‘silva_nr99_v138.1_wSpecies_train_set.fa.gz’ or most updated version and ‘dada2’ R package for taxonomy classification.
    > seq<-rownames(filtered_gut_counts)
    > taxa_clasification <- assignTaxonomy(seq, "data/silva_nr99_v138.1_wSpecies_train_set.fa.gz", multithread=FALSE)
  c. Convert the data into phyloseq object using ‘phyloseq’ R package.
    > asvmat = filtered_gut_counts
    > rownames(asvmat) <- paste0("ASV_", 1:nrow(asvmat))
    > colnames(asvmat) <- paste0("Sample_", 1:ncol(asvmat))
    > taxmat = as.matrix(taxa_clasification[,-1])
    > rownames(taxmat) <- rownames(asvmat)
    > ASV = otu_table(asvmat, taxa_are_rows = TRUE)
    > TAX = tax_table(taxmat)
    > physeq = phyloseq(ASV, TAX)
    > sampledata<-sample_data (data.frame(sample, row.names= sample_names (physeq)))
    > physeq = phyloseq(ASV, TAX, sampledata)
    Example of an expected output of ‘physeq’ R object:

**Figure:**
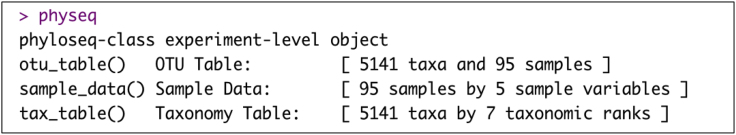
  d. Construct an unrooted phylogenetic tree with random root data using the ‘phangorn’ and ‘DECIPHER’ R packages.
    > seqs<-seq
    > names(seqs) <- rownames(taxmat)
    > alignment <- AlignSeqs(DNAStringSet(seqs), anchor=NA)
    > phang.align <- phyDat(as(alignment, "matrix"), type="DNA")
    > dm <- dist.ml(phang.align)
    > treeNJ <- NJ(dm)
    > fit = pml(treeNJ, data=phang.align)
    > fitGTR <- update(fit, k=4, inv=0.2)
    > fitGTR <- optim.pml(fitGTR, model="GTR", optInv=TRUE, optGamma=TRUE, rearrangement = "stochastic", control = pml.control(trace = 0))
    > physeq_count_with_mit = phyloseq(ASV, TAX, sampledata,phy_tree(fitGTR$tree))
    > set.seed(711)
    > phy_tree(physeq_count_with_mit) <- root(phy_tree(physeq_count_with_mit),sample(taxa_names(physeq_count_with_mit), 1), resolve.root = TRUE)
    Example of an expected output of ‘physeq_count_with_mit’ R object:

**Figure:**
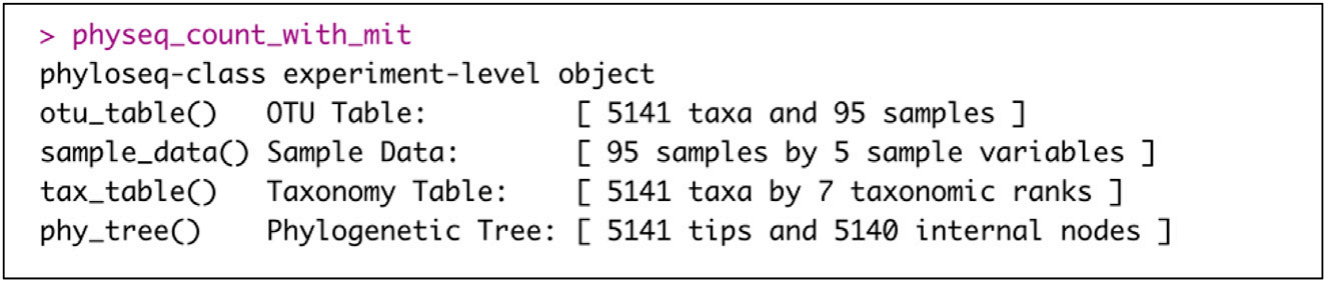
  e. Remov non-bacterial sequences (i.e., mitochondria) from the phyloseq object.
    > MT1 <- subset_taxa(physeq_count_with_mit, Family == "Mitochondria")
    > MT1 <- as(tax_table(MT1), "matrix")
    > MT1 <- MT1[, 5]
    > goodTaxa <- setdiff(taxa_names(physeq_count_with_mit), names(MT1))
    > physeq_count<- prune_taxa(goodTaxa, physeq_count_with_mit)
    Example of an expected output of ‘physeq_count’ R object:

**Figure:**
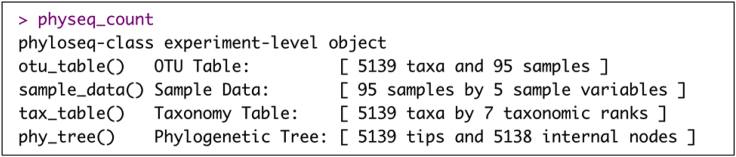
25. Calculate beta diversity measures (i.e., Bray Curtis distance and Unweighted Unifrac distance) and relative abundances for each sample by normalizing counts to 1.

> physeq_ra = transform_sample_counts(physeq_count, function(x) x/sum(x))

26. Inspect the library sizes (i.e., the number of reads) in each sample to identify any heterogeneous library sizes using ‘ggplot2’ and ‘gghighlight’ R packages.

***Note:*** Based on the results, 'Sample 65' is identified with a low library size. However, this sample is not removed from further analysis as there is no compelling reason to do so.

> lib.size <- data.frame(Lib.size=apply(otu_table(physeq_count), 2, sum))

> lib.size$Sample<-rownames(lib.size)

> ggplot(lib.size, aes(x=Sample,y = Lib.size)) +geom_bar(stat = "identity")+ ggpubr::rotate_x_text() +xlab("Sample") + ylab("Library Size")+

gghighlight(min(Lib.size)< 20000, label_key =Sample)

Example of an expected output:

**Figure:**
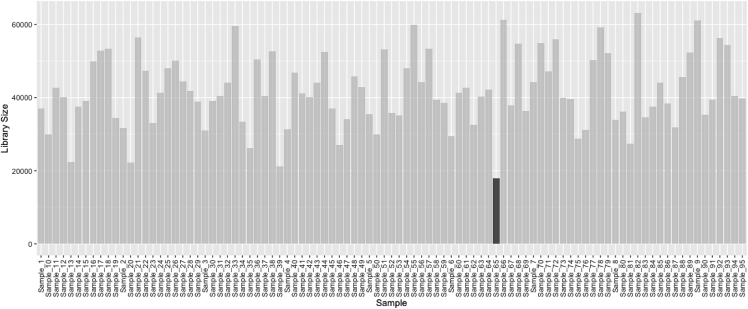

27. Calculate alpha diversity using ‘phyloseq’ R package.
  a. Following Kong et al., 2018 reads are rarefied to 15,000 using ‘phyloseq’ R package. Since the lowest library size is reported to be 17871, all the samples are rarefied to 15,000.
    ***Note:*** The rationale behind rarefaction is to adjust the differences in library sizes across samples to aid comparisons of alpha diversity. However, alpha diversity may not be accurate on rarefied data. Users interested in alpha diversity can normalize samples to a median sequencing depth, which is preferred over rarefaction (McMurdie and Holmes, 2014).
    > physeq.rarefied = rarefy_even_depth(physeq_count, rngseed=1, sample.size=15000, replace=F)
  b. Alpha diversity measures included in Gubert et al. (2022) were:
    i. Species richness (observed).
    ii. Shannon diversity index, which considers richness and relative abundance or evenness of ASVs.
    iii. Inverse Simpson diversity index which also considers richness and relative abundance or evenness of ASVs but is less sensitive to rare species compared to the Shannon index.
      ***Note:*** Other alpha diversity measures are available in ‘estimate_richness’ function (i.e., "Observed", "Chao1", "ACE", "Shannon", "Simpson", "InvSimpson", "Fisher"). If one need all these measures, leave out the argument ‘measures’ (i.e., alpha_div<-estimate_richness(physeq.rarefied)) from the command below.
      > alpha_div<-estimate_richness(physeq.rarefied, measures = c("Observed","Shannon","InvSimpson"))
28. Visualize alpha diversity using boxplots with the use of ‘ggplot2’ and ‘ggpubr’ R packages.

> P1<-plot_richness(physeq.rarefied,x="Housing",measures=c("Observed"), color = "Sex")+facet_grid (∼Genotype)

> P1$layers <- P1$layers[-1]

> P1<-P1+geom_point(position = position_dodge(width=0.75))+geom_boxplot(data = P1$data, aes(x = Housing, y = value, color = Sex),

alpha = 0.1)+ labs(y="Alpha Diversity- Observed")

> P2<-plot_richness(physeq.rarefied, x="Housing",measures=c("Shannon"), color = "Sex")+facet_grid (∼Genotype)

> P2$layers <- P2$layers[-1]

> P2<-P2+geom_point(position = position_dodge(width=0.75))+geom_boxplot(data = P2$data, aes(x = Housing, y = value, color = Sex),

alpha = 0.1)+ labs(y="Alpha Diversity- Shannon")

> P3<-plot_richness(physeq.rarefied, x="Housing",measures=c("InvSimpson"), color = "Sex")+facet_grid (∼Genotype)

> P3$layers <- P3$layers[-1]

> P3<-P3+geom_point(position = position_dodge(width=0.75))+geom_boxplot(data = P3$data, aes(x = Housing, y = value, color = Sex),

alpha = 0.1)+ labs(y="Alpha Diversity- Inverse Simpson")

> ggarrange(P1, P2, P3, labels = c("A", "B", "C"),ncol = 3, nrow = 1)

Example of an expected output:

**Figure:**
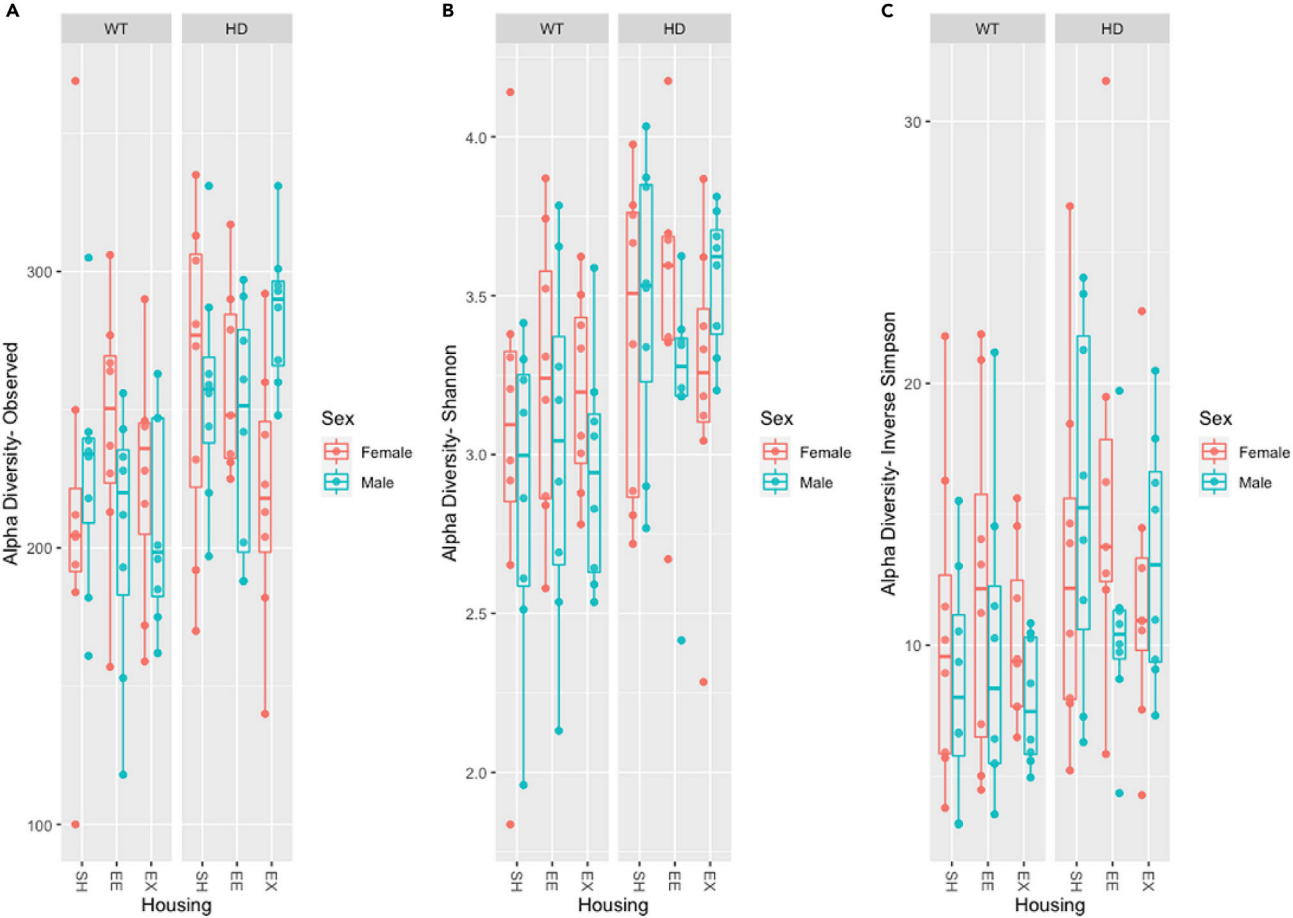

29. Analyze variance of the alpha diversity measures.

> alpha_div.df<-cbind(sample_data(physeq.rarefied)[,1:3],alpha_div)

> summary(aov(Observed∼Housing+Sex+Genotype, data=alpha_div.df))

> summary(aov(Shannon∼Housing+Sex+Genotype, data=alpha_div.df))

> summary(aov(InvSimpson∼Housing+Sex+Genotype, data=alpha_div.df))

Example of an expected output:

**Figure:**
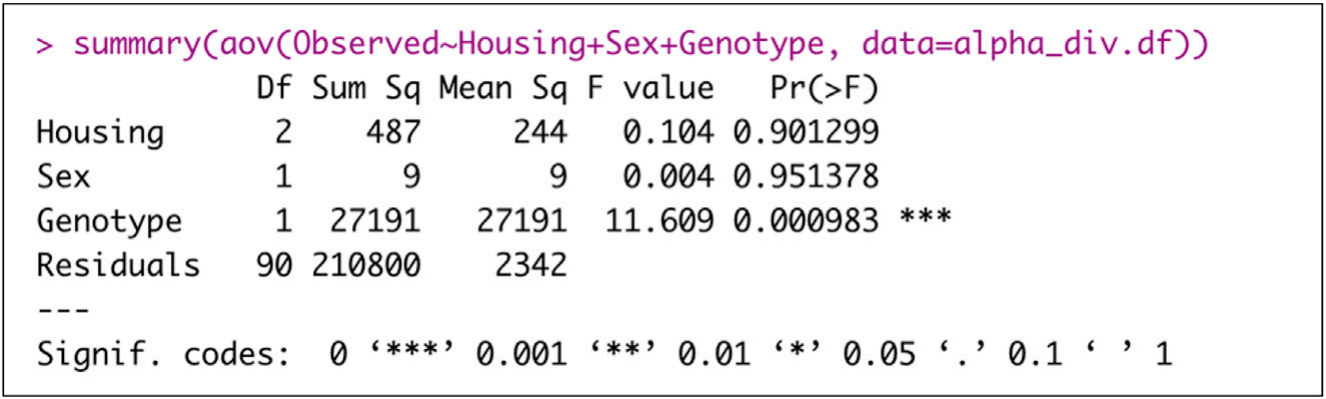

30. Calculate beta diversity using ‘phyloseq’ R package.
  a. Use non rarefied counts.
  b. Bray Curtis distance accounts for the abundance between ASVs.
    > distBC = phyloseq::distance(physeq_ra, method = "bray")
  c. Unweighted Unifrac distance is based on the phylogenetic relationship between the ASVs.
    > distUF = UniFrac(physeq_ra, weighted = F, normalized = T, parallel = F)
31. Visualize beta diversity using Principal Co-ordinate Analysis (PCoA) using ‘phyloseq’ R package.
  a. Visualize PCoA for Bray Curtis distance.
    > bray_pcoa <- ordinate(physeq = physeq_ra, method = "PCoA", distance = "bray"
    > p<-plot_ordination(physeq = physeq_ra, ordination = bray_pcoa,color= "Housing", shape ="Sex_Geno", title = "PCoA: Bray-Curtis")
    > p$layers <- p$layers[-1]
    > p+scale_shape_manual(values = c(16,1,17,2))+ scale_fill_discrete(c("#F8766D","#619CFF","#00BA38"))+ theme(text = element_text(size = 16)) + geom_point(size=4)
    Example of an expected output:

**Figure:**
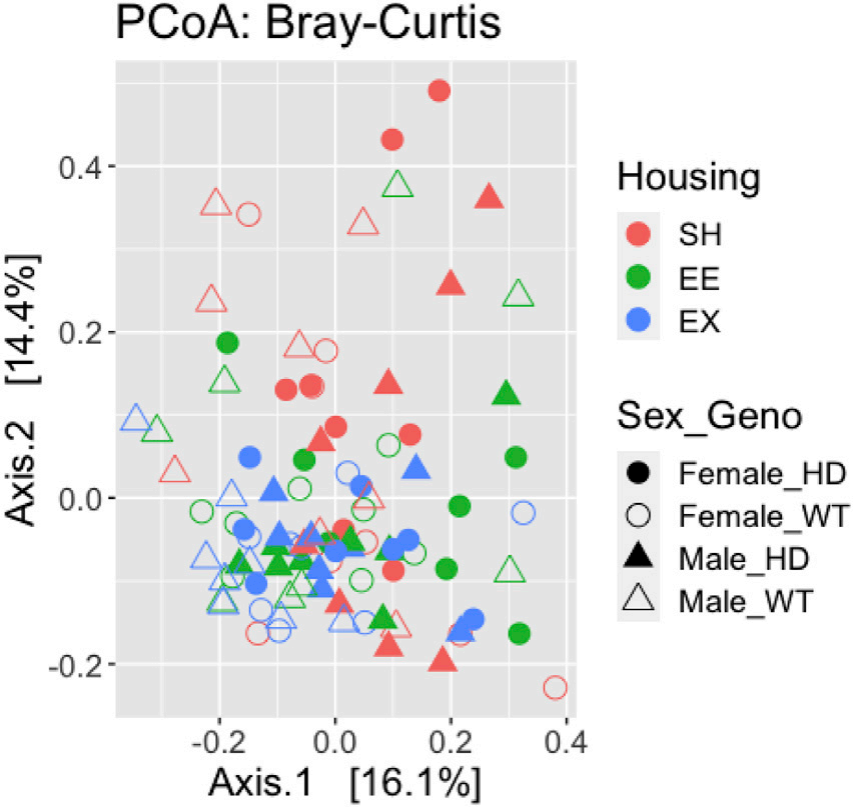
  b. Visulaize PCoA for Unweighted Unifrac distance.
    > ordUF = ordinate(physeq_ra, method = "PCoA", distance = distUF)
    > p<-plot_ordination(physeq_ra, ordUF, color = "Housing", shape = "Sex_Geno")+ ggtitle("Unweighted UniFrac PCoA")
    > p$layers <- p$layers[-1]
    > p+scale_shape_manual(values = c(16,1,17,2)) +
     scale_fill_discrete(c("#F8766D","#619CFF","#00BA38"))+
     theme(text = element_text(size = 16)) + geom_point(size=4)
    Example of an expected output:

**Figure:**
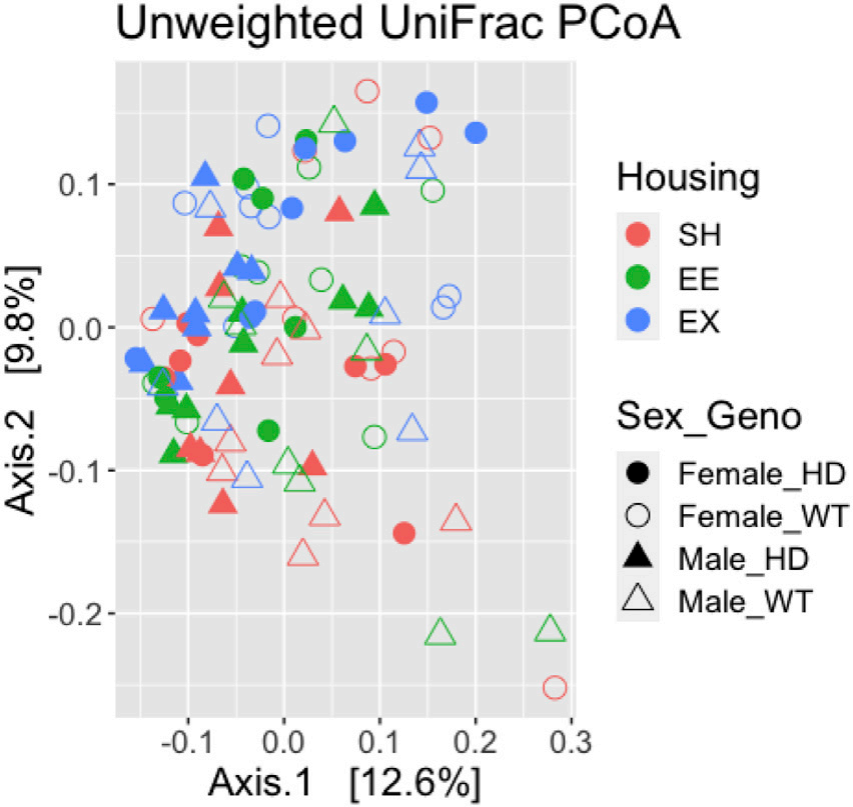
32. Conduct Permutational Multivariate Analysis of Variance (PERMANOVA) on Beta diversity measures using ‘vegan’ R package.
  a. Bray Curtis distance.
    > adonis2(distBC ∼ Housing+Sex+Genotype, data = data.frame(sample_data(physeq_ra)))
    Example of an expected output:

**Figure:**
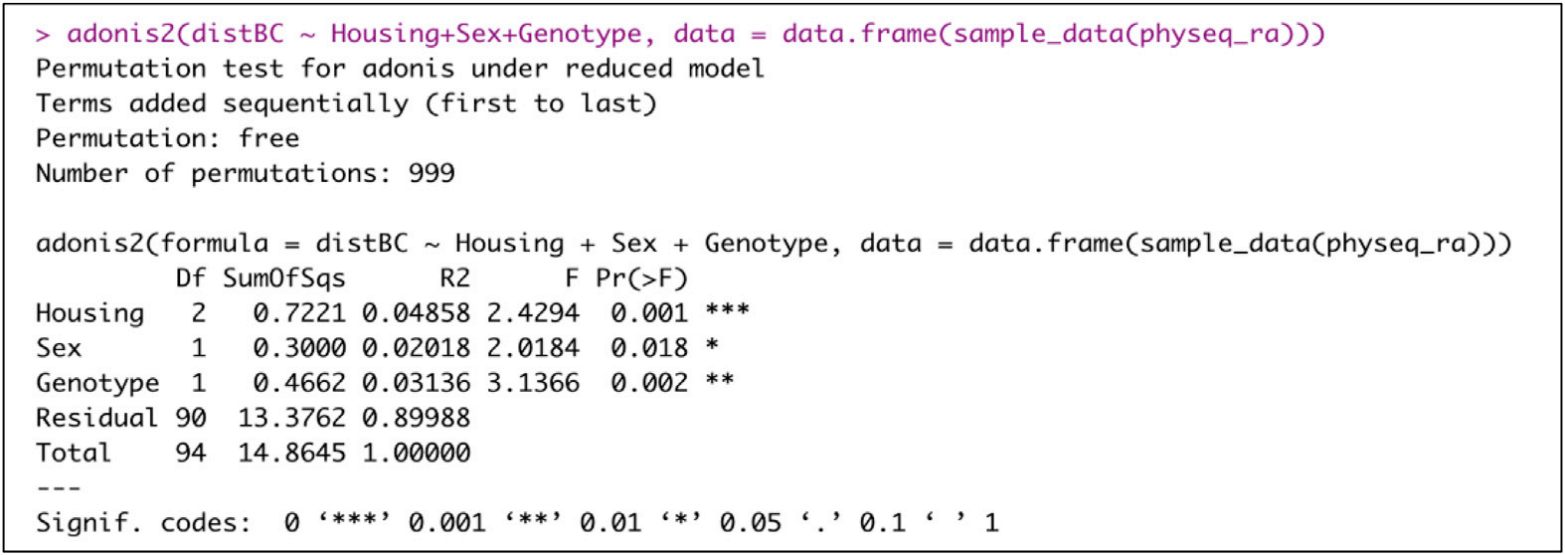
  b. Unweighted Unifrac distance.
    > adonis2(distUF ∼ Housing+Sex+Genotype, data = data.frame(sample_data(physeq_ra)))
    Example of an expected output:

**Figure:**
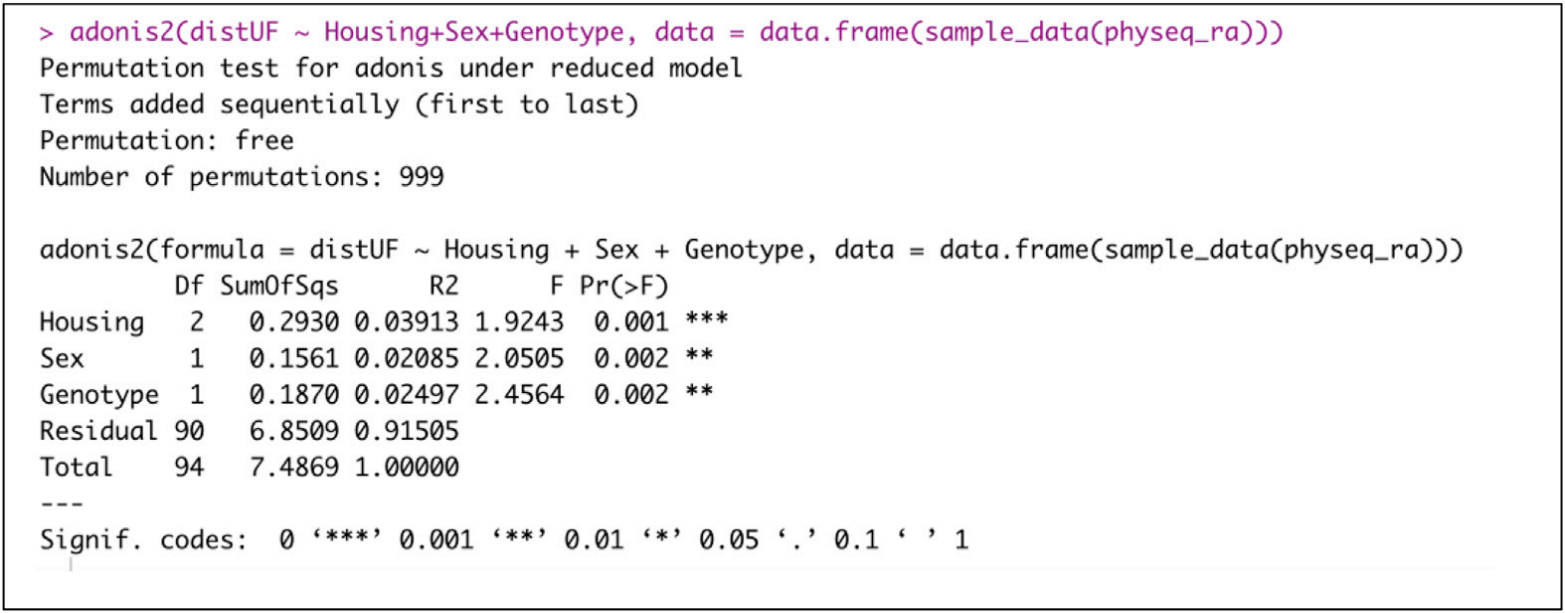
33. Filter counts to remove ASVs with low counts across samples for further statistical analysis:
  a. Add an offset of 1 to the whole data matrix to remove zeros prior to centered log ratio transformation.
    > data.raw = data.frame(t(asvmat))
    > taxonomy.details = data.frame(taxmat)
    > indiv<-data.frame(sample,row.names=rownames(t(asvmat)))
    > Data.16S<-list(data.raw=data.raw+1, taxonomy.details=taxonomy.details, indiv=indiv)
    > data.offset <- Data.16S$data.raw
  b. Remove ASVs with sum counts of less than 0.01% (Le Cao et al., 2016) of the sum of all counts for entire cohort as well as for the two sexes separately.
    > low.count.removal = function(data, percent=0.01){
    keep.asv = which(colSums(data)∗100/(sum(colSums(data))) > percent)
    data.filter = data[,keep.asv]return(list(data.filter = data.filter, keep. asv = keep.oasv))
    }
    ###Entire dataset###
    > result.filter <- low.count.removal(data.offset, percent=0.01) > filter.data.raw<-result.filter$data.filter
    > filter.taxonomy<-taxonomy.details[colnames(filter.data.raw),]
    > filterASV_16S<-list(filter.data.raw=filter.data.raw,
          filter.taxonomy=filter.taxonomy, indiv=indiv)
    ###Females###
    > indiv_female=indiv%>%filter(Sex=="Female")
    > data.raw_female=data.raw[rownames(indiv_female),]
    > taxonomy.female<-taxonomy.details[colnames(data.raw_female),]
    > data.offset.female <- data.raw_female+1
    > result.filter.female <- low.count.removal(data.offset.female, percent=0.01)
    > filter.data.raw.female<-result.filter.female$data.filter
    > filter.taxonomy.female<-taxonomy.details[colnames(filter.data.raw.female),]
    > filterASV_16S.female.pca<-list(filter.data.raw=filter.data.raw.female,
              filter.taxonomy=filter.taxonomy.female, indiv=indiv_female)
    ###Males###
    > indiv_male=indiv%>%filter(Sex=="Male")
    > data.raw_male=data.raw[rownames(indiv_male),]
    > taxonomy.male<-taxonomy.details[colnames(data.raw_male),]
    > data.offset.male <- data.raw_male+1
    > result.filter.male <- low.count.removal(data.offset.male, percent=0.01)
    > filter.data.raw.male<-result.filter.male$data.filter
    > filter.taxonomy.male<-taxonomy.details[colnames(filter.data.raw.male),]
    > filterASV_16S.male.pca<-list(filter.data.raw=filter.data.raw.male,
            filter.taxonomy=filter.taxonomy.male, indiv=indiv_male)
34. Visualize Principal Component Analysis (PCA) results for entire cohort as well as for the two sexes separately using the ‘mixOmics’ R package.

###Entire dataset###

> pca.result <- pca(filterASV_16S$filter.data.raw, logratio = 'CLR')

> plotIndiv(pca.result, group = filterASV_16S$indiv$Housing, title = 'PCA plot', pch.levels =levels(filterASV_16S$indiv$Sex_Geno), pch = c(16,1,17,2), legend = TRUE,ind.names = FALSE,legend.title = 'Housing', legend.title.pch = "Gender_Genotype", col = c("#F8766D","#00BA38","#619CFF"), level.colors=levels(filterASV_16S$indiv$Housing))

###Females###

> pca.result.female <- pca(filterASV_16S.female.pca$filter.data.raw, logratio = 'CLR')

> plotIndiv(pca.result.female, group = filterASV_16S.female.pca$indiv$Housing, pch=filterASV_16S.female.pca$indiv$Genotype,title = 'PCA plot-Female', legend = TRUE, legend.title = "Housing", legend.title.pch = "Genotype", ellipse= TRUE)

###Males###

> pca.result.male <- pca(filterASV_16S.male.pca$filter.data.raw, logratio = 'CLR')

>plotIndiv(pca.result.male, group = filterASV_16S.male.pca$indiv$Housing, pch=filterASV_16S.male.pca$indiv$Genotype,title = 'PCA plot-Male', legend = TRUE, legend.title = "Housing", legend.title.pch = "Genotype",ellipse= TRUE)

Example of an expected output:

**Figure:**
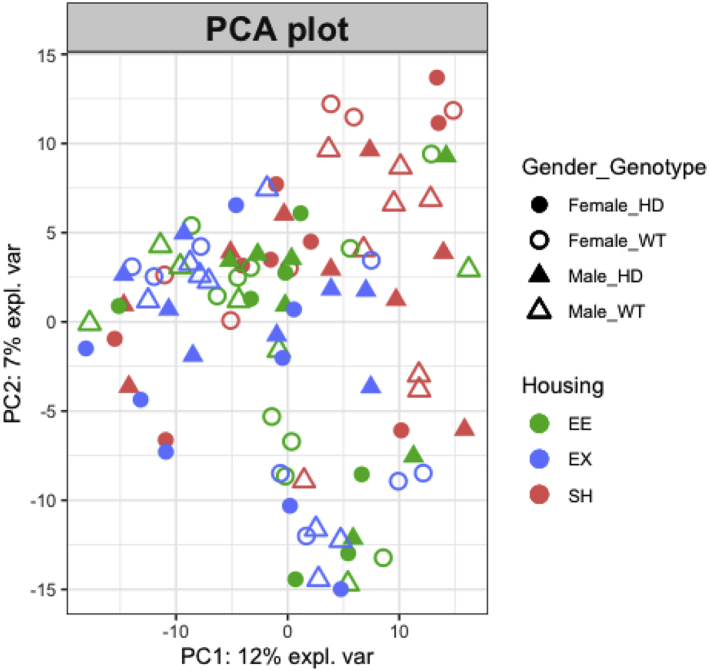

35. Identify ASVs which segregate and contribute to stratification of samples according to housing conditions (for a given sex and genotype) using Sparse Partial Least Squares regression- Discriminant Analysis (sPLS-DA) (Lê Cao et al., 2011) using ‘mixOmics’ and ‘dplyr’ R packages.
  Example of an expected output for the female-HD data.
  a. Choose the optimal parameters for sPLS-DA (number of components and number of variables to select on each component).
    ###Filtering female HD data###
    > female_hd.16S= filterASV_16S.female.pca$indiv%>%filter(Genotype=="HD")
    > Y=female_hd.16S$Housing
    > X=filterASV_16S.female.pca$filter.data.raw[rownames(female_hd.16S),]
    ###Parameter tuning for splsda###
    > set.seed(2543)
    > tune.splsda.female_hd <- tune.splsda(X, Y, ncomp = 3, logratio = 'CLR', validation = 'Mfold', folds = 5, progressBar = FALSE, test.keepX = seq(5,100,5),dist = 'max.dist', measure = "BER", nrepeat = 10)
    > ncomp <- 2
    > select.keepX <- tune.splsda.female_hd$choice.keepX[1:ncomp]
  b. Run sPLS-DA with the optimal parameters, output as 'ncomp' and 'select.keep' from above.
    > splsda.female_hd <- splsda(X, Y, logratio = 'CLR', ncomp = ncomp, keepX = select.keepX)
  c. Visualize sample plots with 0.95 confidence ellipse plots showing discrimination between housing conditions.
    > plotIndiv(splsda.female_hd, group = female_hd.16S$Housing, ind.names = FALSE, ellipse = TRUE, legend = TRUE, title = 'Comp 1 & 2', size.xlabel = rel(1.5), size.ylabel = rel(1.5), size.axis = rel(1), size.legend = rel(1.1), size.legend.title = rel(0), style = "ggplot2")
    Example of an expected output:

**Figure:**
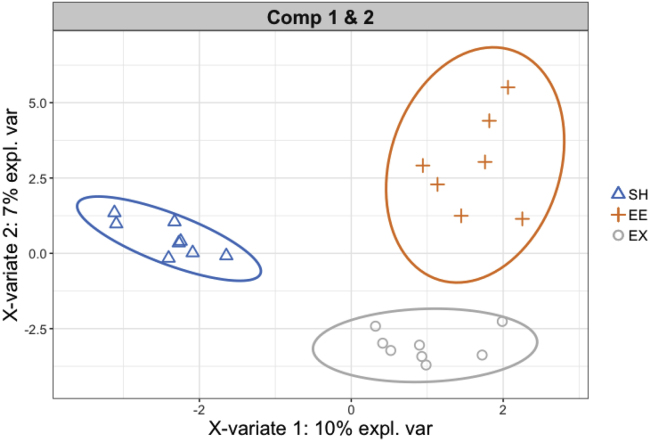
  d. Visualize loading plots showing the highest median value of the ASVs with color code corresponding to the housing conditions.
    > plotLoadings(splsda.female_hd, comp = 1, title = 'Loadings-comp 1', plot = FALSE, contrib = 'max', method = 'median', size.name =0.8,size.title=1)
    > plotLoadings(splsda.female_hd, comp = 2, title = 'Loadings-comp 2', max.name.length=50, plot = FALSE, contrib = 'max', method = 'median', size.name = 1,size.title=1, ndisplay = 35)
    Example of an expected output:

**Figure:**
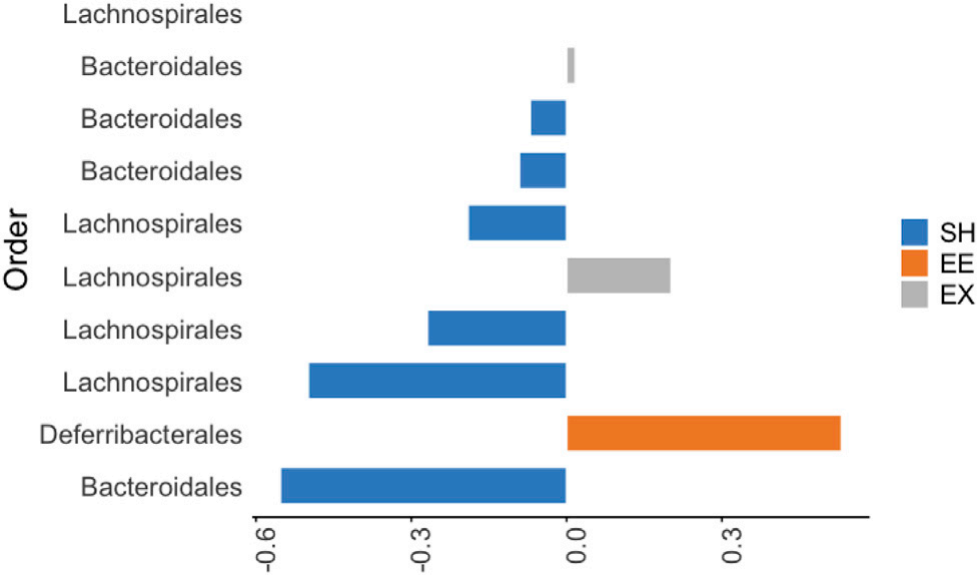
  e. Calculate the overall and class-wise error rates.
    > perf.plsda.female_hd <- perf(splsda.female_hd, validation = "Mfold", folds = 5, progressBar = FALSE, auc = TRUE, nrepeat = 10)
    ### Overall error rates###
    > perf.plsda.female_hd$error.rate
    ### Class-wise error rates###
    **> perf.plsda.female_hd$error.rate.class**
    Example of an expected output:
    i. Overall error rates.

**Figure:**
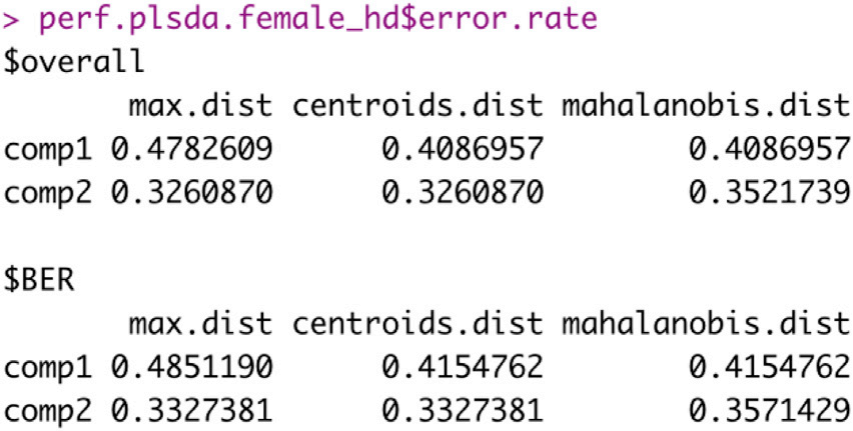
    ii. Class-wise error rates.

**Figure:**
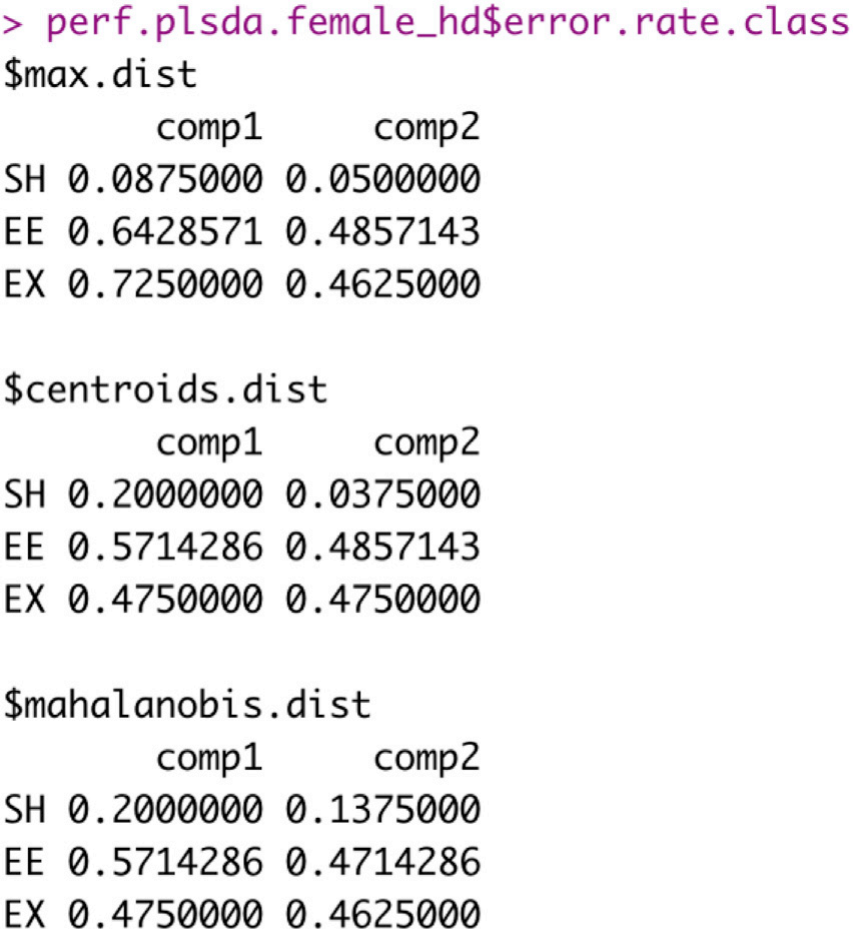
36. Visualize Principal Component Analysis (PCA) results with cage/box number to examine random effects in the data using ‘mixOmics’ R package.
  > plotIndiv(pca.result, group =filterASV_16S$indiv$Box, title = 'PCA plot with Cage', ind.names = filterASV_16S$indiv$Box, cex = 1.5, style = "lattice")
  Example of an expected output:

**Figure:**
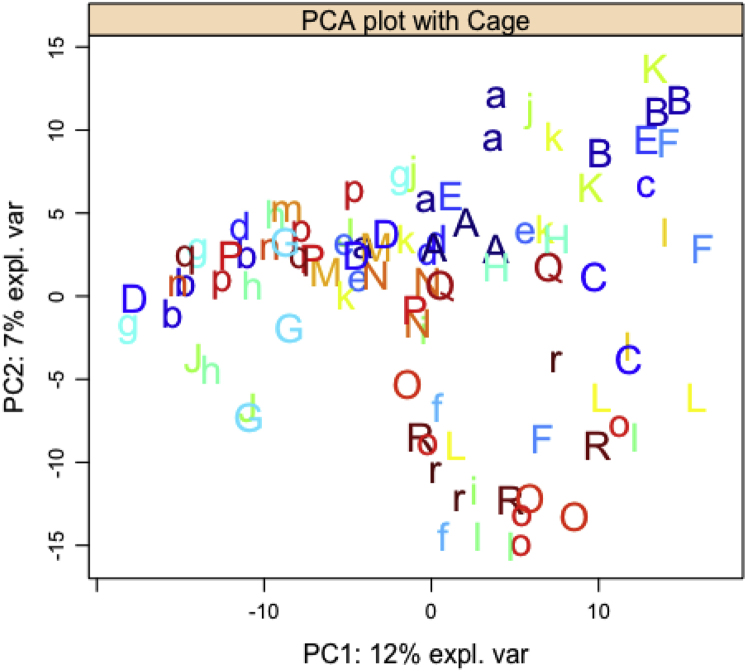
  Since we observed some cage effect, linear mixed models (LMMs) are fitted separately for each of the sexes separately with genotype and housing as fixed effects and cage as random effects to identify which fixed effects and their interactions may explain ASV abundance using ‘nlme’ R package.
  Example for the female data.
  a. To account for compositional data Gloor et al. (2017), first transform raw counts using centred log-ratio transformation.
    > clr.female.data<-logratio.transfo(X = filterASV_16S.female.pca$filter.data.raw, logratio = 'CLR')
    > df.female.clr<-cbind(filterASV_16S.female.pca$indiv, data.frame(clr.female.data[,]))
  b. Fit LMMs on each ASV and extract the relevant p-values. Troubleshooting 6.
    > dep_vars.fe <-grep("ASV_", colnames(df.female.clr), value = T)
    > p_val.fe<-lapply(dep_vars.fe, function(r) {
     f <- formula(paste(r, "Genotype + Housing+ Genotype:Housing", sep = "∼"))
     m <- lme(fixed = f, random = ∼ 1 |Box, data = df.female.clr)
     m$call$fixed <- f
     round(summary(m)$tTable[2:6,5],4)})
    > Matrix_pval.fe <- matrix(unlist(p_val.fe), ncol = 5, byrow = TRUE)
    > rownames(Matrix_pval.fe)<-dep_vars.fe
    > colnames(Matrix_pval.fe)<-c("P-value (HD)", "P-value (EE)", "P-value (EX)", "P-value (HD∗EE)", "P-value (HD∗EX)")
    Example of an expected output:

**Figure:**
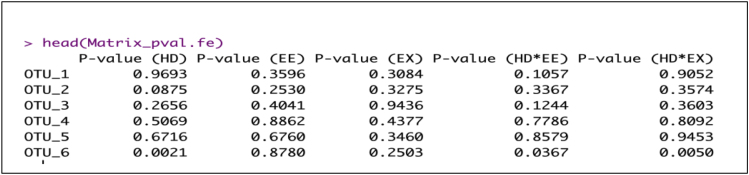
  c. Adjust p-values for multiple testing using the Benjamin and Hochberg (BH) procedure (Benjamini and Hochberg, 1995).
    > Matrix_fdr.fe<-apply(Matrix_pval.fe, 2, function(x) p.adjust(x, method="BH"))
    > colnames(Matrix_fdr.fe)<-c("Adj P-value (HD)", "Adj P-value (EE)", "Adj P-value (EX)", "Adj P-value (HD∗EE)", "Adj P-value (HD∗EX)")
    > female.lmm<-data.frame(Matrix_fdr.fe)
    Example of an expected output:

**Figure:**
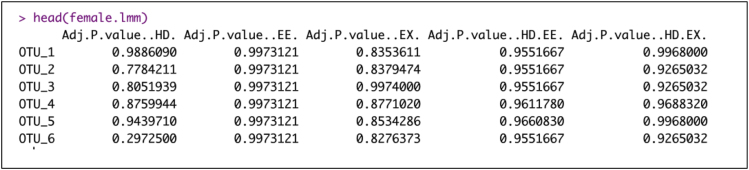

## Expected outcomes

From the DNA extraction, the positive control is expected to yield a DNA concentration of more than 2 ng/μL. The water blank is expected to yield a DNA concentration of close to 0 ng/μL. Fecal samples are expected to yield DNA concentrations of 2–100 ng/μL. Antibiotic treated mice may yield a DNA concentration less than 1 ng/uL. Adjust the elution volume as necessary. The acceptable value of A260/230 and A260/280 ratio should be between 1.8-2.1. Abnormal values for these two ratios may indicate contamination in the extracted sample which may hinder downstream assays. Low concentrations of nucleic acids may result in skewed 260/230 ratio.

When sequencing the 16S amplicon library, loading of 6.5 pM DNA library is expected to obtain the cluster density of 1,000–1,200 K/mm^2^ using MiSeq v2 reagents, although this is highly variable depending on pipetting accuracy when quantitating DNA library concentration or liquid handling. Cluster density of around 800–1,000 K/mm^2^ is also acceptable, lower data output is expected in this case. Both the Q30 and clusters PF should be more than 80%. The generated fastq.gz files are expected to be around 4–5 Gb in total, containing around 24–30 million paired end reads. For 96 samples, the sequencing will generate 125,000–150,000 reads per sample, with the Zymo positive control expected to have around or more than 50,000 reads, while the water negative control is expected to have 0–1,000 reads.

## Limitations

This protocol only refers to studying the bacterial microbiome and does not consider other microbes such as fungi and viruses that also populate the gastrointestinal tract. The 16S amplicon sequencing only assesses bacterial DNA and any other limitations of this kit can be found on the QIAGEN website. Since microbiome data analysis is a fast-moving research area, several methods have been recently proposed, which will improve future research.

The 16S rRNA gene sequences which are used in this protocol are widely used in prokaryotic strains. However, the primers used in the sequencing may be biased toward specific taxa (Martí et al., 2021). Thus, deep amplicon sequencing that includes 18S rRNA gene, internal transcribed spacer for eukaryotes; 16S rRNA and chaperonin-60 genes for bacteria; and Gene 23 and RNA-dependent RNA polymerase for certain viruses, would be more appropriate for a more holistic understanding of the microbiome (Uyaguari-Diaz et al., 2016).

In this protocol, we considered the reference database “silva_nr99_v138.1_wSpecies_train_set.fa.gz” for taxonomic assignment. In addition to SILVA 16S database (Pruesse et al., 2007) there are other databases that can be explored in the future for taxonomy classifications such as Ribosomal Database Project (Cole et al., 2009), Greengenes (DeSantis et al., 2006) and EzTaxon (Chun et al., 2007). However, these reference databases are currently incomplete, resulting in many unclassified sequences at the species level. In addition, there are numerous classification conflicts among these databases (DeSantis et al., 2006).

In the statistical analysis, we compared alpha-diversity measures across samples using rarefied data. However, if the rarefaction curves of the samples do not plateau with respect to library size, it indicates that the sequencing depth and coverage may not be sufficient to cover the total diversity in the gut. Thus, in those scenarios, other normalization methods such as median sequencing depth should be considered (McMurdie and Holmes, 2014).

Since microbiome data are compositional, several authors including Gloor et al. (2017) consider beta-diversity measures, Bray-Curtis and unifrac distances, inappropriate. Thus, recently proposed compositional beta diversity such as Information UniFrac, Ratio UniFrac (Wong et al., 2016) and robust Aitchison PCA (Martino et al., 2019) should be considered instead in future analyses. Using these distances will change the outputs presented in steps 33–35. We also note that PERMANOVA was used to test for microbial divergence among populations. However, other methods such as analysis of group similarities (ANOSIM), multi-response permutation procedures (MRPP), and Mantel’s test (MANTEL) can also be used to test group differences in microbiome data (Xia and Sun, 2017).

Due to the cage effects in the data, we only use LMMs to identify which effects may explain ASV abundance. As discussed in Nearing et al. (2022), one should use multiple methods to ensure robust biological inferences when the sample size is large enough. For instance, if the analytical objective is to test for differential abundance then methods such as edgeR (Robinson et al., 2010), DESeq2 (Love et al., 2014), ANCOM (Mandal et al., 2015) or ZIGDM can be applied (Tang and Chen, 2019). Additionally, if the objective is to infer correlated taxa, then methods such as SparCC (Friedman and Alm, 2012), CCLasso (Fang et al., 2015), REBACCA (Ban et al., 2015), SpiecEasi (Kurtz et al., 2015), HARMONIES (Jiang et al., 2020) and SPRING (Yoon et al., 2019) can be used. There are also several statistical methods developed for longitudinal microbiome data (Kodikara et al., 2022).

## Troubleshooting

### Problem 1

Urine in fecal samples.

Mice may urinate within the sterilized cage during fecal collection, which could potentially contaminate fecal pellets with the urobiome.

### Potential solution

Ensure that no urine has contaminated the fecal pellets by only collecting feces that has not come in to contact with urine, via visual inspection of the area around each fecal pellet.

### Problem 2

Low DNA yield from extraction.

Low DNA concentration during the extraction process may be observed in samples from mice that underwent antibiotic treatment, or when low amounts of raw fecal material was used for this process.

### Potential solution

Use more raw fecal material during the extraction process or reduce the elution volume.

### Problem 3

Potential PCR Contamination.

Genomic DNA contamination from other sources during PCR amplification can cause inaccurate or unreliable results.

### Potential solution

Physically separate pre and post PCR areas within the lab, ensuring separate sinks, water purification systems, protocol supplies, equipment and storage spaces. Also make sure all PCR areas are cleaned daily using 80% ethanol to reduce risk of contamination. Ensure that a water blank is processed along with the samples for quality control. Taxa identified in the samples which were also found in water control post-sequencing and denoising can be removed from the analysis.

### Problem 4

DNA does not amplify during PCR.

Samples may have low library yield post-PCR which may stem from lows DNA input or contamination in the extracted DNA which can inhibit PCR amplification. Excess amounts of DNA may also inhibit PCR reactions.

### Potential solution

Make sure to check the DNA yield using fluorometric methods such as Qubit or Quant-iT assay. The purity of the extracted DNA should be checked using the spectrophotometer. If the A260/230 or A260/280 ratio is abnormal, DNA clean-up using SPRI beads can be performed. If DNA concentration is too high, the extracted DNA can be diluted with the elution buffer.

### Problem 5

Low number of sequencing reads.

Samples may have low read counts post sequencing. This may stem from inaccurate DNA quantification of each library for normalization or when quantifying the library pool for loading onto the sequencer resulting in low sequencing output.

### Potential solution

Ensure that fluorometric assays such as Qubit or Quant-iT kit are used for DNA quantification. Accurate pipetting techniques should be employed here as well. Samples may also be quantitated in replicates to improve accuracy.

### Problem 6

Data interpretation in high-dimensional data.

16S sequencing produces high-dimensional data that are harder to interpret.

### Potential solution

Data visualizations in projection-based methods (i.e., PCA, sPLS-DA) used in this protocol support data interpretation. For example, in step 36, the PCA plot visualizes the samples colored according to cages to detect potential cage effects in the data. Methods such as LMM produce p-values which can be used to infer significant genotype and housing effects on ASV abundance among the two sexes separately.

## Resource availability

### Lead contact

Further information and requests for resources and reagents should be directed to and will be fulfilled by the lead contact, Anthony J. Hannan (anthony.hannan@florey.edu.au).

### Materials availability

This study did not generate new unique reagents.

## Data Availability

The datasets and metadata related to this study have been deposited in the NCBI Sequence Read Archive. The accession number for the raw sequence reads reported in this paper is BioProject number PRJNA770470. The reproducible R code and report for the statistical analysis has been uploaded to a GitHub repository - https://github.com/SarithaKodikara/Gene_environment_gut_interactions_in_Huntington-s_disease. An archived version of the GitHub repository is available at Zenodo: https://zenodo.org/badge/latestdoi/416519533.
